# Supplementary material for: KMSubtraction: reconstruction of unreported subgroup survival data utilizing published Kaplan-Meier survival curves
Source: BMC Med Res Methodol. 2022 Apr 3;22:93. doi: 10.1186/s12874-022-01567-z (PMC8978435; doi:10.1186/s12874-022-01567-z)
Supplement: Supplementary file 1 — Additional file 1. [file 12874_2022_1567_MOESM1_ESM.docx]

**SUPPLEMENTARY MATERIALS**

**KMSubtraction: Reconstruction of unreported subgroup survival data utilizing published Kaplan-Meier survival curves**

Joseph J. Zhao^1^; Nicholas L. Syn^1^, MBBS; Benjamin Kye Jyn Tan^1^; Dominic Wei Ting Yap^1^; Chong Boon Teo^1^; Yiong Huak Chan^2,^ *, PhD; Raghav Sundar^1, 3, 4, 5, 6,^ *, MBBS, PhD

^1^ Yong Loo Lin School of Medicine, National University of Singapore, Singapore

^2^ Biostatistics Unit, Yong Loo Lin School of Medicine, National University of Singapore, Singapore

^3^ Department of Haematology-Oncology, National University Health System, Singapore

^4^ Cancer and Stem Cell Biology Program, Duke-NUS Medical School, Singapore

^5^ The N.1 Institute for Health, National University of Singapore, Singapore

^6^ Singapore Gastric Cancer Consortium

* Joint corresponding author

**Supplementary Guide**

The vignette is made available on github: [https://github.com/josephjzhao/KMSubtraction](https://github.com/josephjzhao/KMSubtraction#readme)

**Supplementary Table 1** Simulation parameters

The independent variable (denoted by red boxes) follows according to the following arithmetic sequence: $\lim_{n\to m} (a+(1-n)*b)$

| **Variable being investigated** | **Size of overall cohort (*N*_overall_)** | **Proportion of reported subgroup (*P*_subgroup_)** | **Proportion of patients with censorship in overall cohort**  **(*P*_overall, censored_)** | **Proportion of patients with censorship in subgroup cohort**  **(*P*_subgroup, censored_)** | **Proportion of missing data (*P*_missing_)** | **Number of number-at-risk table intervals**  **(*N*_interval_)** | **Number of Monte Carlo iterations**  **(i)** |
| --- | --- | --- | --- | --- | --- | --- | --- |
| ***N*_overall_** | $a=50,$  $b=1000,$  $m=20$ | 0.50 | 0.50 | 0.50 | 0.00 | 8 | 5000 |
| ***P*_subgroup_** | 500 | $a=0.01,$  $b=0.05,$  $m=20$ | 0.50 | 0.50 | 0.00 | 8 | 5000 |
| ***P*_overall, censored_** | 500 | 0.50 | $a=0.01,$  $b=0.05,$  $m=20$ | * | 0.00 | 8 | 5000 |
| ***P*_subgroup, censored_** | 500 | 0.50 | * | $a=0.01,$  $b=0.05,$  $m=20$ | 0.00 | 8 | 5000 |
| ***P*_missing_** | 500 | 0.50 | 0.50 | 0.50 | $a=0.01,$  $b=0.05,$  $m=19$ | 8 | 5000 |
| ***N*_interval_** | 500 | 0.50 | 0.50 | 0.50 | 0.00 | $a=2,$  $b=1,$  $m=19$ | 5000 |

* Since the subgroups are dichotomous, the relationship between *P*_overall, censored_ and *P*_subgroup, censored_ is bounded by

$\frac{Psubgroup*Poverall, censored}{Psubgroup, censored}=\frac{Nsubgroup, censored}{Noverall, censored}\leq1$

Thus, when the proportion of censored patients from the overall and subgroup cohorts were investigated as the independent variable, we arbitrarily defined $\frac{Nsubgroup, censored}{Noverall, censored}=0.5$_._

This gives $Psubgroup, censored=\frac{P\mathrm{subgroup}*Poverall, censored}{0.5}$ _and_ $Poverall, censored=\frac{0.5*Psubgroup, censored}{P\mathrm{subgroup}}$

when the proportion of censored patients from the overall and subgroup cohorts were investigated respectively.

**Supplementary Table 2** Significant outcomes of Tukey multiple pairwise-comparisons between each matching algorithm

| **Comparison of matching algorithms** | **Parameters** | | | | | | **Difference (95% CI)** | **p** |
| --- | --- | --- | --- | --- | --- | --- | --- | --- |
|  | **Size of overall cohort (*N*_overall_)** | **Proportion of reported subgroup (*P*_subgroup_)** | **Proportion of patients with censorship in overall cohort**  **(*P*_overall, censored_)** | **Proportion of patients with censorship in subgroup cohort**  **(*P*_subgroup, censored_)** | **Proportion of missing data (*P*_missing_)** | **Number of number-at-risk table intervals**  **(*N*_interval_)** |  |  |
| **Logistic/Bipartite** | 500 | 0.86 | 0.50 | 0.50 | 0.00 | 8 | 0.003166297 (0.001300484 to 0.005032110) | **<0.001** |
| **Mahalanobis/Logistic** | 500 | 0.86 | 0.50 | 0.50 | 0.00 | 8 | -0.002886512 (-0.004752325 to -0.001020700) | **<0.001** |
| **Logistic/Bipartite** | 500 | 0.91 | 0.50 | 0.50 | 0.00 | 8 | 0.005700769 ( 0.003834957 to 0.007566582) | **<0.001** |
| **Mahalanobis/Logistic** | 500 | 0.91 | 0.50 | 0.50 | 0.00 | 8 | -0.007242428 (-0.009108240 to -0.005376615) | **<0.001** |
| **Logistic/Bipartite** | 500 | 0.96 | 0.50 | 0.50 | 0.00 | 8 | 0.010563824 (0.008698011 to 0.012429636) | **<0.001** |
| **Mahalanobis/Bipartite** | 500 | 0.96 | 0.50 | 0.50 | 0.00 | 8 | -0.017536851 (-0.019402664 to -0.015671039) | **<0.001** |
| **Mahalanobis/Logistic** | 500 | 0.96 | 0.50 | 0.50 | 0.00 | 8 | -0.028100675 (-0.029966488 to -0.026234863) | **<0.001** |

Abbreviations: Logistic, nearest neighbor matching with distances defined by logistic regression; Bipartite, minimal cost bipartite matching; Mahalanobis, Mahalanobis distance matching; CI, confidence interval.

**Supplementary Figure 1** Running mean plots demonstrating simulation convergence


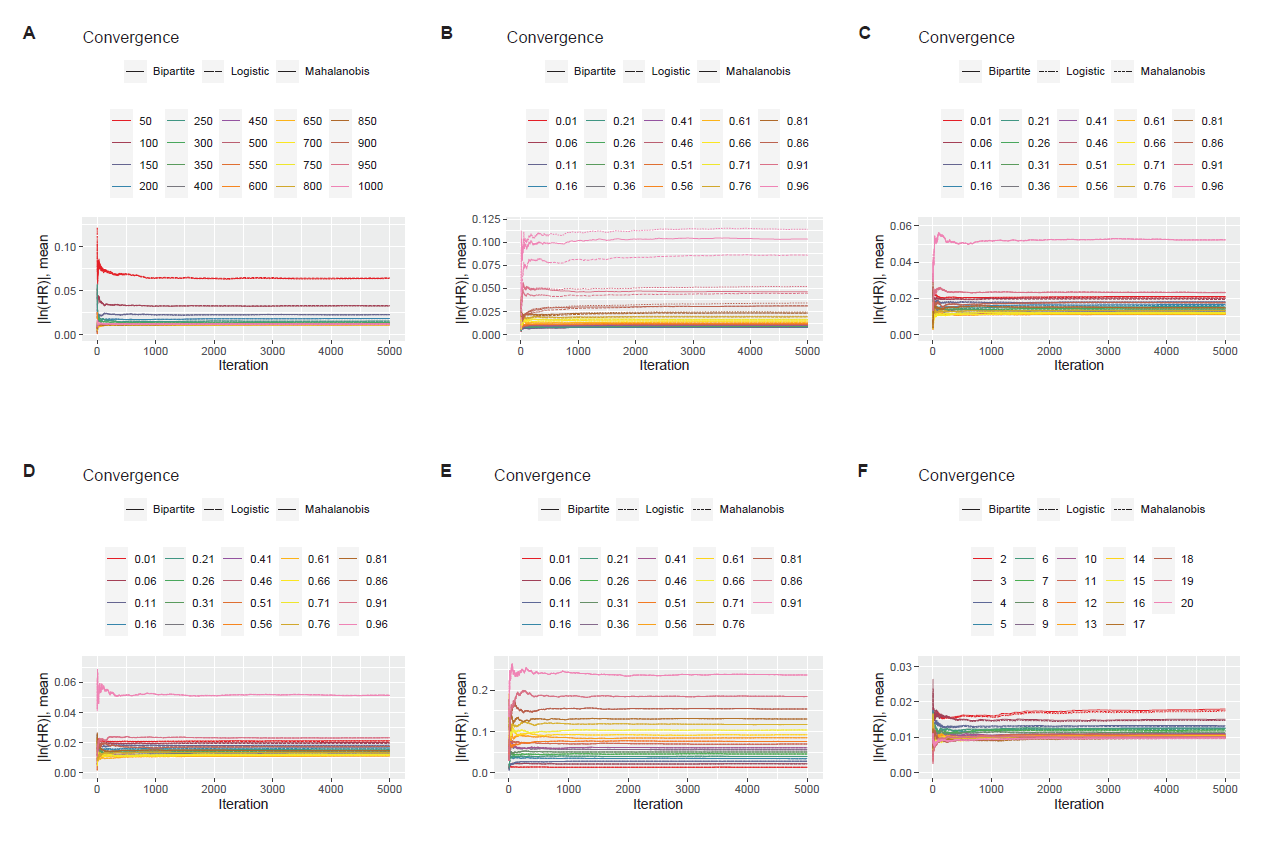


Running mean plots demonstrating simulation convergence for (A) Size of dataset (B) Proportion of reported subgroup (C) Proportion of patients with censorship in the overall cohort (D) Proportion of patients with censorship in the subgroup cohort (E) Proportion of missing data (F) Number of number-at-risk table intervals

**Supplementary Code**

# Implemented in R-4.1.0

main_wd="C:/Users/jzhao/OneDrive/Research_Cloud/NUH_NCIS/KMSubtraction/"

setwd(main_wd)

# number of Monte Carlo iterations per scenario

n.mc=5000

# set number of cores

detectCores()

n.cores=6

#### DOWNLOAD libraries ####

package.name=c(# unique to KMSubtraction

"MatchIt", "wakefield", "magick", "RcppHungarian", "scales", "IPDfromKM", "svglite", "rsvg", "blandr", "KMSubtraction",

# data manipulation

"tidyverse", "reshape", "readr", "readxl", "dplyr", "tidyr", "lubridate", "tibble", "plyr", "devtools", "stringr", "stringi",

# parallel processing

"doParallel", "parallel", "foreach", "doSNOW",

# survival analysis

"survminer", "survival",

# misc

"cluster", "ResourceSelection", "progress"

)

for (package.name in package.name){

if (!require(package.name, character.only = TRUE)){

install.packages(package.name, character.only = TRUE)

library(package.name, character.only = TRUE)} else {library(package.name, character.only = TRUE)}}

#### LOAD functions ####

seq_sim=function(x){seq(from=as.numeric(str_extract(x, "^(\\d|\\.)*(?=;)")),

to=as.numeric(str_extract(x, "(?<=;)(\\d|\\.)*(?=;)")),

as.numeric(str_extract(x, "(?<=;)(\\d|\\.)*$")))}

#### LOAD Simulation parameters ####

simulation_parameters=data.frame(outcome = c("outcome_size", "outcome_subgroup", "outcome_censorship_overall", "outcome_censorship_subgroup", "outcome_missing", "outcome_interval"),

n = c("50;1000;50", "500;500;0", "500;500;0", "500;500;0", "500;500;0", "500;500;0"),

subgroup.p = c("0.5;0.5;0", "0.01;0.99;0.05", "0.5;0.5;0", "0.5;0.5;0", "0.5;0.5;0", "0.5;0.5;0"),

censor_overall.p = c("0.5;0.5;0", "0.5;0.5;0", "0.01;0.99;0.05","0.5;0.5;0", "0.5;0.5;0", "0.5;0.5;0"),

censor_subgroup.p = c("0.5;0.5;0", "0.5;0.5;0", "0.5;0.5;0", "0.01;0.99;0.05", "0.5;0.5;0", "0.5;0.5;0"),

missing.p = c("0;0;0", "0;0;0", "0;0;0", "0;0;0", "0.01;0.91;0.05", "0;0;0"),

interval = c("8;8;0", "8;8;0", "8;8;0", "8;8;0", "8;8;0", "2;20;1"),

mc = rep(paste0("1;", n.mc,";1" ), 6))

# count the total number of iterations

iterations=0

for (r in 1:nrow(simulation_parameters)){

row.iterations=1

for (c in 2:ncol(simulation_parameters)){

row.iterations=row.iterations*length(seq_sim(simulation_parameters[r,c]))

}

iterations=row.iterations+iterations

}

print(paste0("Total number of iterations: ", iterations))

set.seed(NULL)

#### Prepare parallel processing settings ####

registerDoParallel(n.cores)

registerDoSNOW(makeSOCKcluster(n.cores))

# progress bar

pb <- txtProgressBar(min=1, max=iterations, style=3)

progress <- function(n) setTxtProgressBar(pb, n)

opts <- list(progress=progress)

#### Simulation ####

df_out=NULL

i.outcome=1

system.time({

df_out=

foreach (i.outcome = 1:length(simulation_parameters$outcome), .options.snow=opts, .combine=rbind) %:%

foreach (i.n = 1:length(seq_sim(simulation_parameters$n[i.outcome])), .options.snow=opts, .combine=rbind) %:%

foreach (i.censor_overall = 1:length(seq_sim(simulation_parameters$censor_overall.p[i.outcome])), .options.snow=opts, .combine=rbind) %:%

foreach (i.censor_subgroup = 1:length(seq_sim(simulation_parameters$censor_subgroup.p[i.outcome])), .options.snow=opts, .combine=rbind) %:%

foreach (i.subgroup = 1:length(seq_sim(simulation_parameters$subgroup.p[i.outcome])), .options.snow=opts, .combine=rbind) %:%

foreach (i.missing = 1:length(seq_sim(simulation_parameters$missing.p[i.outcome])), .options.snow=opts, .combine=rbind) %:%

foreach (i.interval = 1:length(seq_sim(simulation_parameters$interval[i.outcome])), .options.snow=opts, .combine=rbind) %:%

foreach (i.mc = 1:length(seq_sim(simulation_parameters$mc[i.outcome])), .options.snow=opts, .combine=rbind) %dopar% {

library(survival)

library(survminer)

library(wakefield)

library(svglite)

library(magick)

library(stringi)

library(stringr)

library(dplyr)

library(tidyr)

library(tidyverse)

library(scales)

library(svglite)

library(rsvg)

library(IPDfromKM)

library(readr)

library(RcppHungarian)

library(dplyr)

library(MatchIt)

library(survRM2)

library(blandr)

library(KMSubtraction)

#### 0_Define parameters ####

outcome=simulation_parameters$outcome[i.outcome]

# assumptions and settings

mc=seq_sim(simulation_parameters$mc[i.outcome])

n=seq_sim(simulation_parameters$n[i.outcome])

censor_overall.p=seq_sim(simulation_parameters$censor_overall.p[i.outcome])

censor_subgroup.p=seq_sim(simulation_parameters$censor_subgroup.p[i.outcome])

missing.p=seq_sim(simulation_parameters$missing.p[i.outcome])

subgroup.p=seq_sim(simulation_parameters$subgroup.p[i.outcome])

interval=seq_sim(simulation_parameters$interval[i.outcome])

#### 1_Create simulation data ####

if (outcome=="outcome_censorship_overall"){

censor_subgroup.p[i.censor_subgroup]=(subgroup.p[i.subgroup]*censor_overall.p[i.censor_overall])/0.5

}

if (outcome=="outcome_censorship_subgroup"){

censor_overall.p[i.censor_overall]=(0.5*censor_subgroup.p[i.censor_subgroup])/(subgroup.p[i.subgroup])

}

# number of patients per subgroup

n.subgroup=round(subgroup.p[i.subgroup]*n[i.n],0)

n.opposingsubgroup=round((n[i.n]-n.subgroup),0)

# number of censored patients in the opposing subgroup

n.censoroverall=round(censor_overall.p[i.censor_overall]*n[i.n], 0)

n.censorsubgroup=round(censor_subgroup.p[i.censor_subgroup]*n.subgroup,0)

n.censoroppsingsubgroup=n.censoroverall-n.censorsubgroup

censor_opposingsubgroup.p=n.censoroppsingsubgroup/n.opposingsubgroup

# generating simulated data

df_overall=NULL

df_overall$time=rweibull(n.subgroup+n.opposingsubgroup, shape=1,scale=5)

df_overall$status=c(rep(0, n.censorsubgroup),

rep(1, n.subgroup-n.censorsubgroup),

rep(0, n.censoroppsingsubgroup),

rep(1, n.opposingsubgroup-n.censoroppsingsubgroup))

df_overall$subgroup=c(rep(1, n.subgroup),

rep(0, n.opposingsubgroup))

df_overall$subgroup[df_overall$subgroup==0]=r_sample_binary(length(df_overall$subgroup[df_overall$subgroup==0]), x = c(NA,0), prob = c(missing.p[i.missing], 1-missing.p[i.missing]), name = "Binary")

df_overall=data.frame(df_overall)

df_subgroup=subset(df_overall, df_overall$subgroup==1)

group=paste0("_n",n[i.n],

"_subgroup", subgroup.p[i.subgroup],

"_censoroverall", censor_overall.p[i.censor_overall],

"_censorsubgroup", censor_subgroup.p[i.censor_subgroup],

"_missing", missing.p[i.missing],

"_interval", interval[i.interval],

"_mc", mc[i.mc])

if(nrow(df_subgroup)==0){

stop("Subgroup=0")

}

km_overall=survfit(Surv(time, status) ~ 1, data=df_overall)

km_subgroup=survfit(Surv(time, status) ~ 1, data=df_subgroup)

df_overall_clicks=getxycoordinates(df_overall, label=paste0("overall",group))

df_subgroup_clicks=getxycoordinates(df_subgroup, label=paste0("subgroup",group))

#### 2_Reconstruction ####

# at risk tables

df_overall_risktable=ggsurvtable(km_overall, data=df_overall, break.time.by = (max(df_overall$time)/interval[i.interval]))$risk.table$data[,2:3]

df_subgroup_risktable=ggsurvtable(km_subgroup, data=df_subgroup, break.time.by = (max(df_subgroup$time)/interval[i.interval]))$risk.table$data[,2:3]

# overall

df_overall_recon=getIPD(prep=preprocess(dat=df_overall_clicks,

trisk=df_overall_risktable$time,

nrisk=df_overall_risktable$n.risk,

maxy=1),

armID=1, tot.events=NULL)$IPD

# subgroup

df_subgroup_recon=getIPD(prep=preprocess(dat=df_subgroup_clicks,

trisk=df_subgroup_risktable$time,

nrisk=df_subgroup_risktable$n.risk,

maxy=1),

armID=1, tot.events=NULL)$IPD

#### 3_Matching ####

match.algo=c("bipartite", "maha", "logit")

tbl=NULL

for (i.match in match.algo){

df_match=KMSubtractionMatch(df_overall_recon, df_subgroup_recon, matching=i.match)$data

df_match$strata="matched"

df_overall$strata="original"

df_combined=bind_rows(df_match, df_overall)

# survival analysis

sum.cox=coxph(formula = Surv(time, status) ~ strata, data=subset(df_combined, df_combined$subgroup==0)) %>% summary

GT=coxph(formula = Surv(time, status) ~ strata, data=subset(df_combined, df_combined$subgroup==0)) %>% cox.zph

# collate

tbl=rbind(tbl,c(outcome=outcome,

n=n[i.n],

subgroup.p=subgroup.p[i.subgroup],

censorship_overall.p=censor_overall.p[i.censor_overall],

censorship_subgroup.p=censor_subgroup.p[i.censor_subgroup],

missing.p=missing.p[i.missing],

interval=interval[i.interval],

mc=mc[i.mc],

logrank=sum.cox$sctest[3],

HR=sum.cox$coefficients[2],

TE=sum.cox$coefficients[1],

se=sum.cox$coefficients[3],

GT.p=GT$table[1,3],

matching=i.match))

}

#### 4_Export ####

tbl

}

})

# clear parallel processing

stopImplicitCluster()

close(pb)

stopCluster(makeSOCKcluster(n.cores))

#### Export as csv ####

# cleaning

df_out=data.frame(df_out)

df_out[,-grep("matching|outcome", colnames(df_out))]=apply(df_out[,-grep("matching|outcome", colnames(df_out))],2, as.numeric)

# write

write.csv(df_out, paste0("df_run.csv"))
